# Supplementary material for: STING signalling is terminated through ESCRT-dependent microautophagy of vesicles originating from recycling endosomes
Source: Nat Cell Biol. 2023 Mar 13;25(3):453–66. doi: 10.1038/s41556-023-01098-9 (PMC10014584; doi:10.1038/s41556-023-01098-9)
Supplement: Supplementary file 1 — Reporting Summary [file 41556_2023_1098_MOESM1_ESM.pdf]

nature portfolio

Corresponding author(s): Tomohiko Taguchi

Last updated by author(s): Jan 15, 2023

Reporting Summary

Nature Portfolio wishes to improve the reproducibility of the work that we publish. This form provides structure for consistency and transparency in reporting. For further information on Nature Portfolio policies, see our Editorial Policies and the Editorial Policy Checklist.

Statistics

For all statistical analyses, confirm that the following items are present in the figure legend, table legend, main text, or Methods section.

n/a	Confirmed	
☐	☒	The exact sample size (*n*) for each experimental group/condition, given as a discrete number and unit of measurement
☐	☒	A statement on whether measurements were taken from distinct samples or whether the same sample was measured repeatedly
☐	☒	The statistical test(s) used AND whether they are one- or two-sided<br>*Only common tests should be described solely by name; describe more complex techniques in the Methods section.*
☐	☒	A description of all covariates tested
☐	☒	A description of any assumptions or corrections, such as tests of normality and adjustment for multiple comparisons
☐	☒	A full description of the statistical parameters including central tendency (e.g. means) or other basic estimates (e.g. regression coefficient) AND variation (e.g. standard deviation) or associated estimates of uncertainty (e.g. confidence intervals)
☐	☒	For null hypothesis testing, the test statistic (e.g. *F*, *t*, *r*) with confidence intervals, effect sizes, degrees of freedom and *P* value noted<br>*Give P values as exact values whenever suitable.*
☒	☐	For Bayesian analysis, information on the choice of priors and Markov chain Monte Carlo settings
☒	☐	For hierarchical and complex designs, identification of the appropriate level for tests and full reporting of outcomes
☐	☒	Estimates of effect sizes (e.g. Cohen's *d*, Pearson's *r*), indicating how they were calculated

Our web collection on statistics for biologists contains articles on many of the points above.

Software and code

Policy information about availability of computer code

Data collection	Western blot data were collected using FUSION SOLO (software;Evolution Capt)<br>Microscopy data were collected using Zeiss ZEN 2.3 SP1 FP3 (black, 64 bit) (ver. 14.0.21.201)<br>CLEM data were collected sing JEM1400EX; JEOL.<br>The data of the luciferase activity was collected by GloMax® Navigator Microplate Luminometer (Promega) (version 3.1.0).<br>Quantitative real-time PCR (qRT-PCR) was performed using LightCycler 96 (Roche).
Data analysis	Western blot data were analysed by Fiji (ver. 2.1.0/1.53c).<br>Microscopy data were analysed by Fiji (ver. 2.1.0/1.53c) including the Trainable Weka Segmentation plugin (v3.3.2), Cellpose (v1.0), R (ver. 4.1.2), and KNIME (ver. 4.5.1).<br>Proteomics data were analysed by MASCOT (ver. 2.6), Proteome Discover (ver. 2.2).

For manuscripts utilizing custom algorithms or software that are central to the research but not yet described in published literature, software must be made available to editors and reviewers. We strongly encourage code deposition in a community repository (e.g. GitHub). See the Nature Portfolio guidelines for submitting code & software for further information.

Data

Policy information about availability of data

All manuscripts must include a data availability statement. This statement should provide the following information, where applicable:

- Accession codes, unique identifiers, or web links for publicly available datasets
- A description of any restrictions on data availability
- For clinical datasets or third party data, please ensure that the statement adheres to our policy

The authors declare that the data supporting the findings of this study are available within the supplementary information. NCBI nr database is available in (https://www.ncbi.nlm.nih.gov). Mass spectrometry data have been deposited in ProteomeXchange with the primary accession code PXD039411.

Field-specific reporting

Please select the one below that is the best fit for your research. If you are not sure, read the appropriate sections before making your selection.

☒ Life sciences ☐ Behavioural & social sciences ☐ Ecological, evolutionary & environmental sciences

For a reference copy of the document with all sections, see nature.com/documents/nr-reporting-summary-flat.pdf

Life sciences study design

All studies must disclose on these points even when the disclosure is negative.

Sample size	No sample size calculation was applied in this study to predetermine sample sizes for experiments using cell lines. A sample size of three or more was used as to evaluate the spread of the data and was determined based upon other studies with similar methodologies (PMID: 27324217, 29093443, 33397928)
Data exclusions	No data have been excluded from any analysis.
Replication	All experiments have been repeated at least three times independently, and each yielding similar results.
Randomization	Randomization was not relevant for cell culture study, because all cells used in this study had to be differently treated and analyzed in paralle to minimize experimental variation.
Blinding	All the experiments were unblinded because these experiments were not susceptible to bias.

Reporting for specific materials, systems and methods

We require information from authors about some types of materials, experimental systems and methods used in many studies. Here, indicate whether each material, system or method listed is relevant to your study. If you are not sure if a list item applies to your research, read the appropriate section before selecting a response.

Materials & experimental systems

n/a	Involved in the study	
☐	☒	Antibodies
☐	☒	Eukaryotic cell lines
☒	☐	Palaeontology and archaeology
☐	☒	Animals and other organisms
☐	☒	Human research participants
☒	☐	Clinical data
☒	☐	Dual use research of concern

Methods

n/a	Involved in the study	
☒	☐	ChIP-seq
☐	☒	Flow cytometry
☒	☐	MRI-based neuroimaging

Antibodies

Antibodies used	Antibodies used in this study were as follows: anti-Atg5 (MBL Life science, PM050, dilution 1/1000, WB), anti-STING (proteintech, 19851-1-AP, dilution 1/1000, WB), anti-tubulin (Sigma-Aldrich, DM1A, dilution 1/1000, WB), anti-GM130 (BD Biosciences, 610823, dilution 1/1000, IF), anti-Rab11 (Cell Signaling Technology, D4F5, dilution 1/100, IF), anti-GAPDH (MERCK, 6C5, dilution 1/1000, WB), anti-pTBK1 (Cell Signaling Technology, D52C2, dilution 1/1000, IF/WB), anti-TBK1 (Abcam, ab40676, dilution 1/1000, WB), anti-ubiquitin(P4D1) (Abcam, ab139101, dilution 1/1000, WB), anti-K63 ubiquitin (millipore, 05-1308, dilution 1/100, IF), anti-GFP (Clontech, JL-8, dilution 1/1000, WB), anti-GFP (Thermo Fisher Scientific, 3E6, dilution 1/500, IP), anti-Goat Anti-Rabbit IgG (H+L) Mouse/Human ads-HRP (Southern Biotech, 4050-05, dilution 1/1000, WB), anti-Goat Anti-Mouse IgG (H+L) Human ads-HRP (Southern Biotech, 1031-05, dilution 1/1000, WB), anti-Alexa 488-, 594-, or 647- conjugated secondary antibodies (Thermo Fisher Scientific, A21202, A21203, A21206, A21207, A31573, A11016, A21448, dilution 1/1000, IF). anti-LC3 (MBL Life science, PM036

	1/1000, IF/WB), anti-EEA1 (BD Biosciences, 610456, 1/500 IF), anti-LBPA (Merck Millipore, 6C4, 1/500, IF), anti-Clathrin heavy chain (Cell Signaling Technology, D3C6 ,1/1000 IF), anti-Tsg101 (Abcam, ab125011, 1/500, WB), anti-Lamp1 (eBioscience, 1D4B, 1/1000, IF)
Validation	All antibodies were validated by the vendors and documented with corresponding data sheets as follows.<br>mouse anti-Atg5 (MBL Life science, PM050): validated for mouse Atg5 by WB with cell lysate.<br>rabbit anti-STING (proteintech, 19851-1-AP): validated for mouse STING by WB with cell lysate.<br>mouse anti-tubulin (Sigma-Aldrich, DM1A): validated for mouse tubulin by WB with cell lysate.<br>mouse anti-GM130 (BD Biosciences, 610823): validated for mouse GM130 by IF with fixed cells.<br>rabbit anti-Rab11 (Cell Signaling Technology, D4F5): validated for mouse Rab11 by IF with fixed cells.<br>rabbit anti-GAPDH (MERCK, 6C5): validated for mouse GAPDH by WB with cell lysate.<br>rabbit anti-pTBK1 (Cell Signaling Technology, D52C2): validated for mouse pTBK1 by IF with fixed cells and by WB with cell lysate.<br>rabbit anti-TBK1 (Abcam, ab40676): validated for mouse TBK1 by WB with cell lysate.<br>mouse anti-ubiquitin(P4D1) (Abcam, ab139101): validated for mouse ubiquitin(P4D1) by WB with cell lysate.<br>rabbit anti-K63 ubiquitin (millipore, 05-1308): validated for mouse K63 ubiquitin by IF with fixed cells.<br>mouse anti-GFP (Clontech, JL-8): validated for mouse GFP by WB with cell lysate.<br>mouse anti-GFP (Thermo Fisher Scientific, 3E6): validated for mouse GFP by IP with western blotting.<br>Goat Anti-Rabbit IgG (H+L) Mouse/Human ads-HRP (Southern Biotech, 4050-05): validated for mouse Goat Anti-Rabbit IgG (H+L) Mouse/Human ads-HRP by WB with cell lysate.<br>Goat Anti-Mouse IgG (H+L) Human ads-HRP (Southern Biotech, 1031-05): validated for mouse Goat Anti-Mouse IgG (H+L) Human ads-HRP by WB with cell lysate.<br>donkey Alexa 488-, 594-, or 647- conjugated secondary antibodies (Thermo Fisher Scientific, A21202, A21203, A21206, A21207, A31573, A11016, A21448): validated for mouse Alexa 488-, 594-, or 647- conjugated secondary antibodies by IF with fixed cells.<br>rabbit anti-LC3 (MBL Life science, PM036): validated for mouse LC3 by WB and IF with fixed cells.<br>mouse anti-EEA1 (BD Biosciences, 610456): validated for mouse EEA1 by IF with fixed cells.<br>mouse anti-LBPA (Merck Millipore, 6C4): validated for mouse LBPA by IF with fixed cells.<br>rabbit anti-Clathrin heavy chain (Cell Signaling Technology, D3C6) : validated for mouse Clathrin heavy chain by IF with fixed cells.<br>rabbit anti-Tsg101 (Abcam, ab125011, 1/500, WB): validated for mouse/human Tsg101 by WB.<br>mouse anti-Lamp1 (eBioscience, 1D4B, 1/1000, IF): validated for mouse Lamp1 by WB.

Eukaryotic cell lines

Policy information about cell lines

Cell line source(s)	HEK293T cells were from ATCC. Immortalized MEFs were described in (PMID: 27324217). Vero cells were described in (PMID: 32994400). Raw264.7 were from InvivoGen. MRC-5 cells were from the Riken BioResource Center.
Authentication	Authentication of HEK293T cells was performed by ATCC with the short tandem repeat profiling. MEFs were identified by genotyping. Authentication of MRC-5 cells was performed by the Riken BioResource Center with the short tandem repeat profiling. No methods was used for authentication for Raw264.7.
Mycoplasma contamination	Confirm that all cell lines were tested negative for mycoplasma contaminations.
Commonly misidentified lines (See ICLAC register)	No commonly misidentified cell lines were used in the study.

Animals and other organisms

Policy information about studies involving animals; ARRIVE guidelines recommended for reporting animal research

Laboratory animals	C57BL/6 mice
Wild animals	This study does not involve wild animals.
Field-collected samples	This study does not involve field-collected samples.
Ethics oversight	Ethics number PA17-84 approved by the Institute of Medical Sciences of the University of Tokyo.

Note that full information on the approval of the study protocol must also be provided in the manuscript.

Human research participants

Policy information about studies involving human research participants

Population characteristics	Healthy human samples were collected from four Japanese males aged 30 - 55 with no significant medical history.
Recruitment	Participants were recruited from individuals working at Pediatric department of Kyoto University Hospital by word of mouth.
Ethics oversight	All experiments involving human subjects were conducted in accordance with the principles of the Declaration of Helsinki and were approved by the ethics committee of Kyoto University Hospital (protocol number: G1233). Written informed consent was obtained from the participants before sampling. No compensation was provided.

Note that full information on the approval of the study protocol must also be provided in the manuscript.

Flow Cytometry

Plots

Confirm that:

- [ ] The axis labels state the marker and fluorochrome used (e.g. CD4-FITC).
- [ ] The axis scales are clearly visible. Include numbers along axes only for bottom left plot of group (a 'group' is an analysis of identical markers).
- [ ] All plots are contour plots with outliers or pseudocolor plots.
- [ ] A numerical value for number of cells or percentage (with statistics) is provided.

Methodology

Sample preparation	Sting-/- MEFs reconstituted with mRuby3-STING were treated with indicated siRNA for 54 h followed by stimulation with or without DMXAA for 18 h. Cells were detached with trypsin/EDTA and fixed with 4% PFA in PBS at room temperature for 15 min.
Instrument	Cell Sorter SH800 (Sony)
Software	Software version 2.1(Cell Sorter SH800 (Sony))
Cell population abundance	*Describe the abundance of the relevant cell populations within post-sort fractions, providing details on the purity of the samples and how it was determined.*
Gating strategy	No gating for FSC/SSC. No gating for the signal of mRuby3.

- [ ] Tick this box to confirm that a figure exemplifying the gating strategy is provided in the Supplementary Information.
