# Supplementary figures and images for: STING signalling is terminated through ESCRT-dependent microautophagy of vesicles originating from recycling endosomes
Source: Nat Cell Biol. 2023 Mar 13;25(3):453–66. doi: 10.1038/s41556-023-01098-9 (PMC10014584; doi:10.1038/s41556-023-01098-9)

**Fig. 6a**

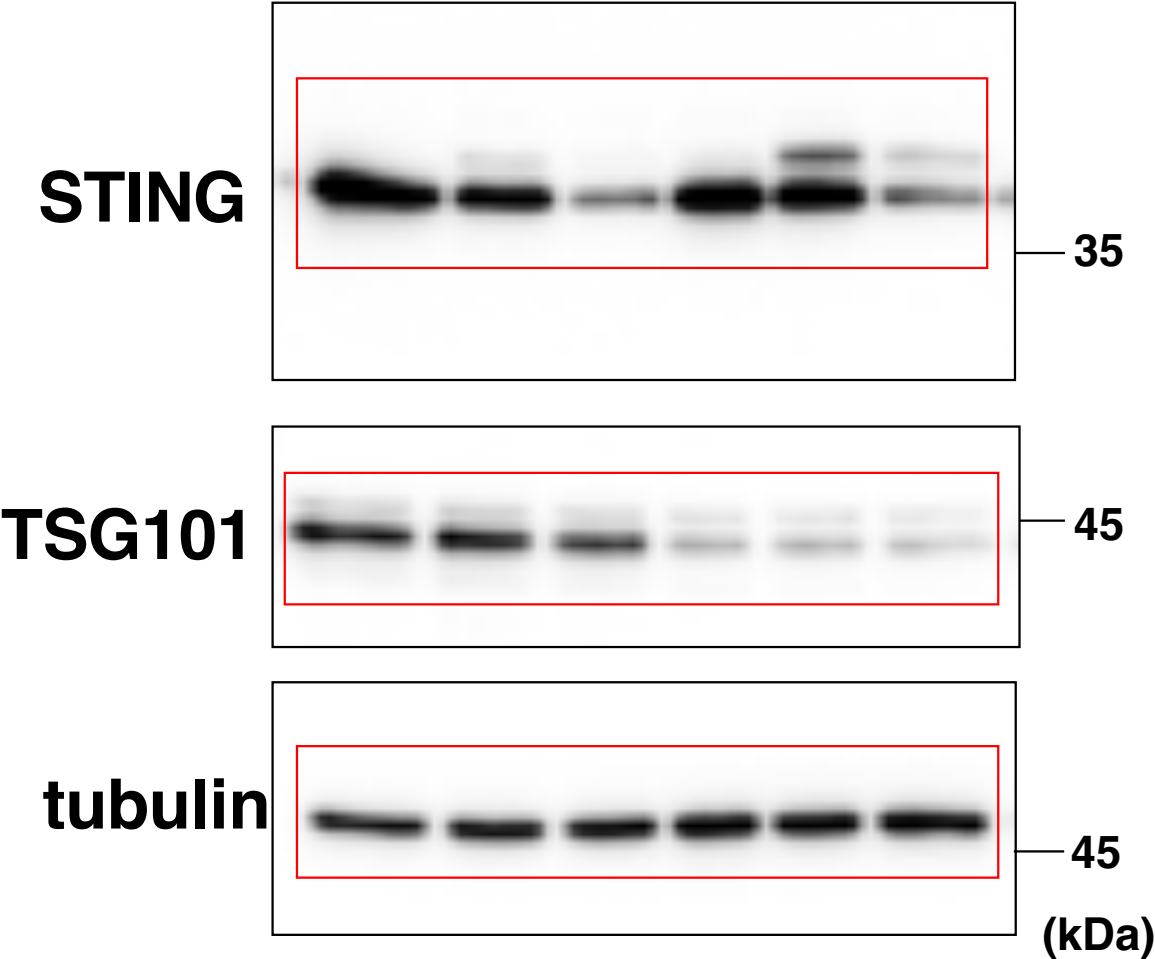

**Fig. 6g**

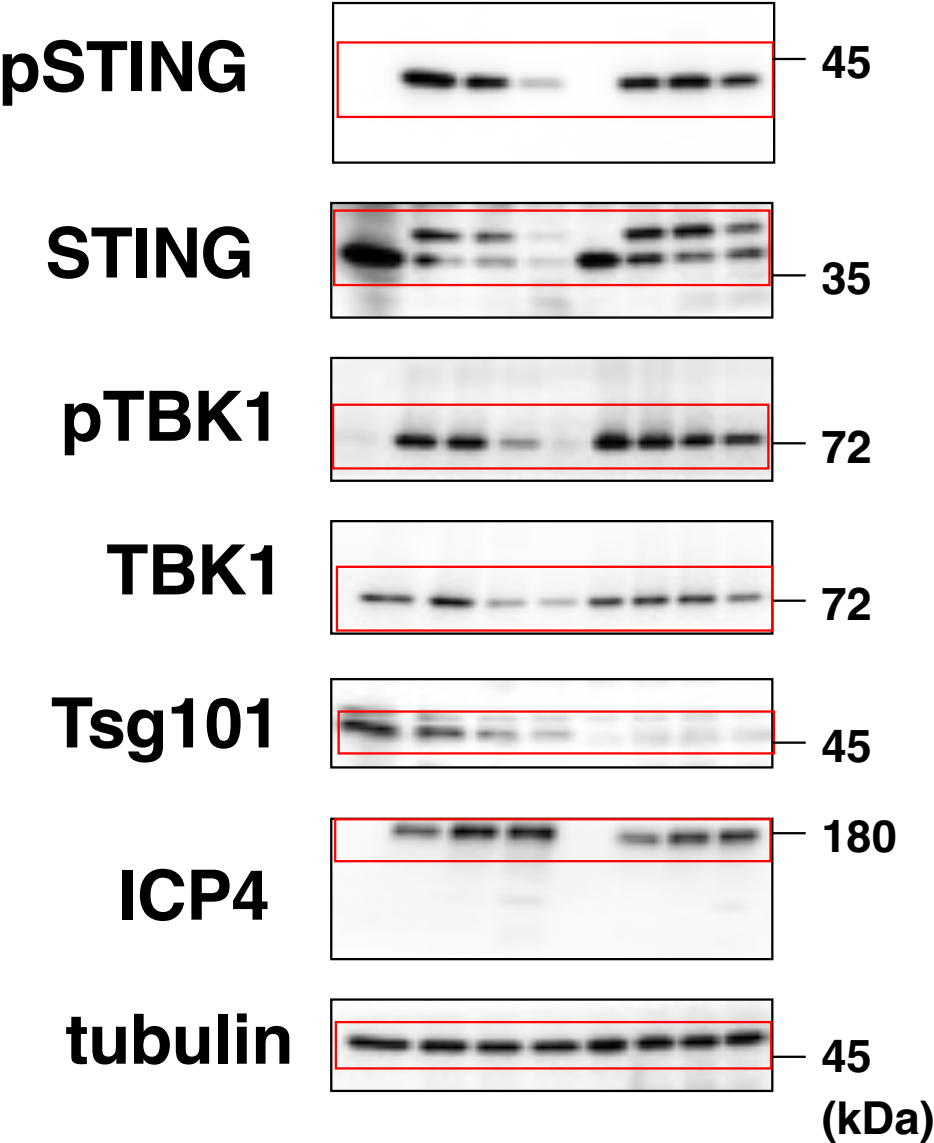

Supplement: Source Data Fig. 6 — Unprocessed western blots and/or gels. [file 41556_2023_1098_MOESM12_ESM.pdf]

**Fig. 7a**

**IP:GFP, ubiquitin**

**GFP**

**pTBK1**

**TBK1**

**tubulin**

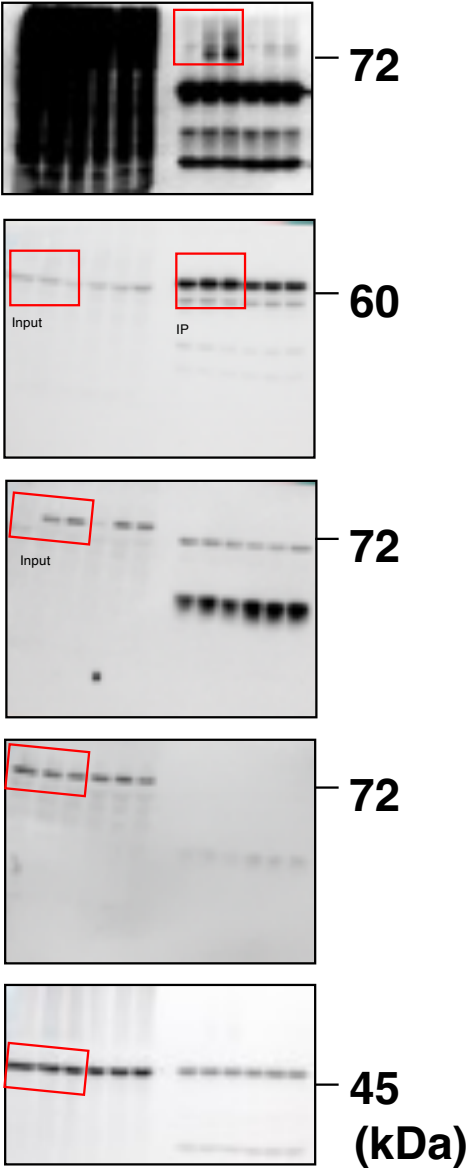

**Fig. 7d**

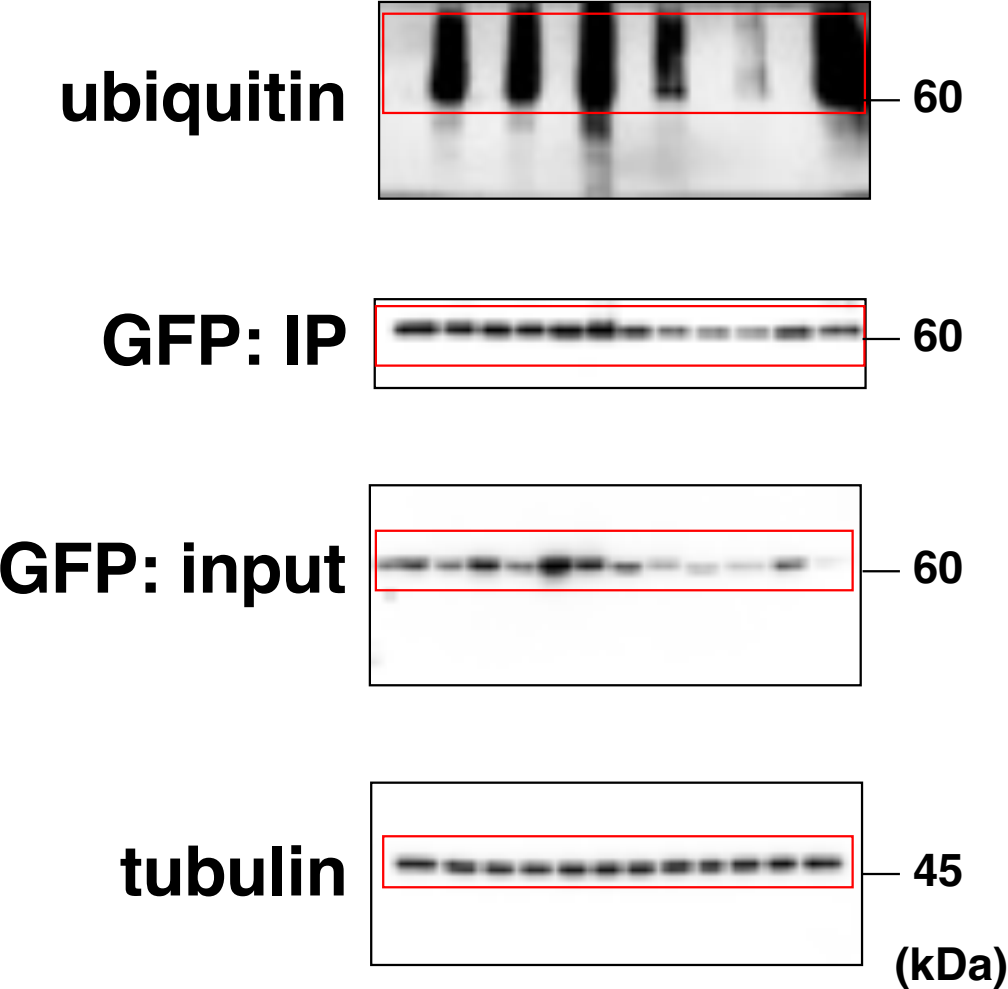

**Fig. 7g**

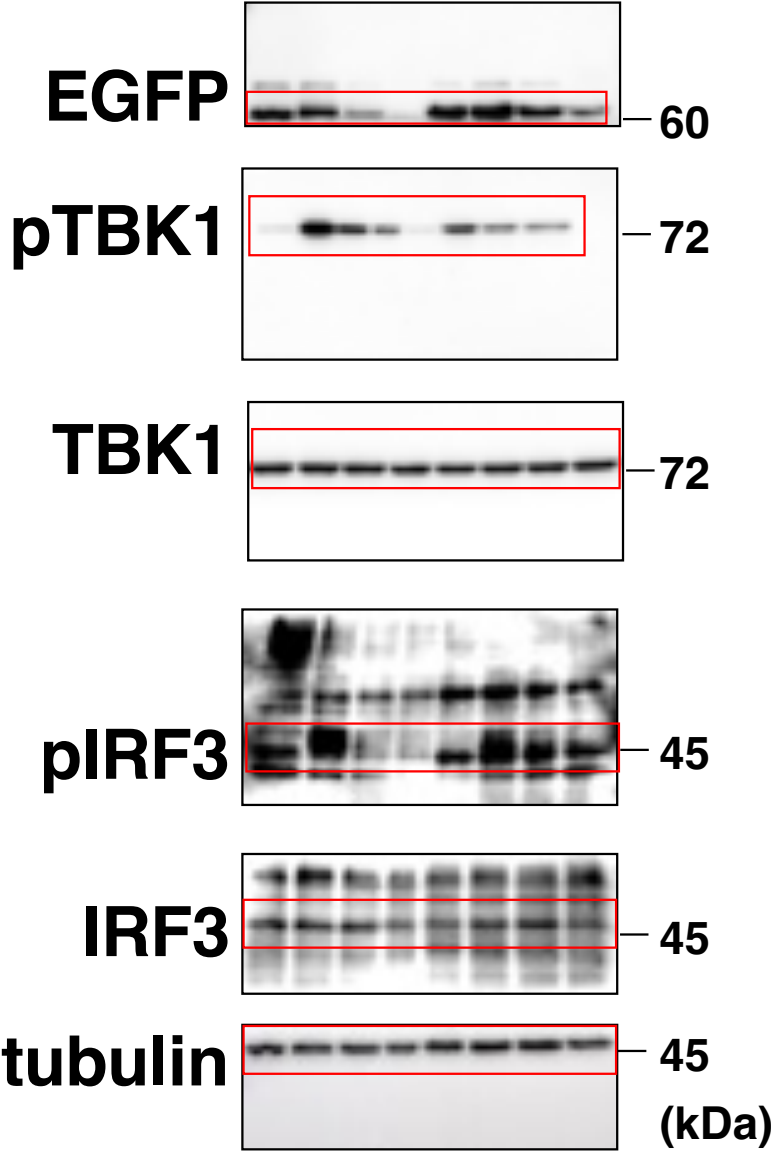

**Fig. 7k**

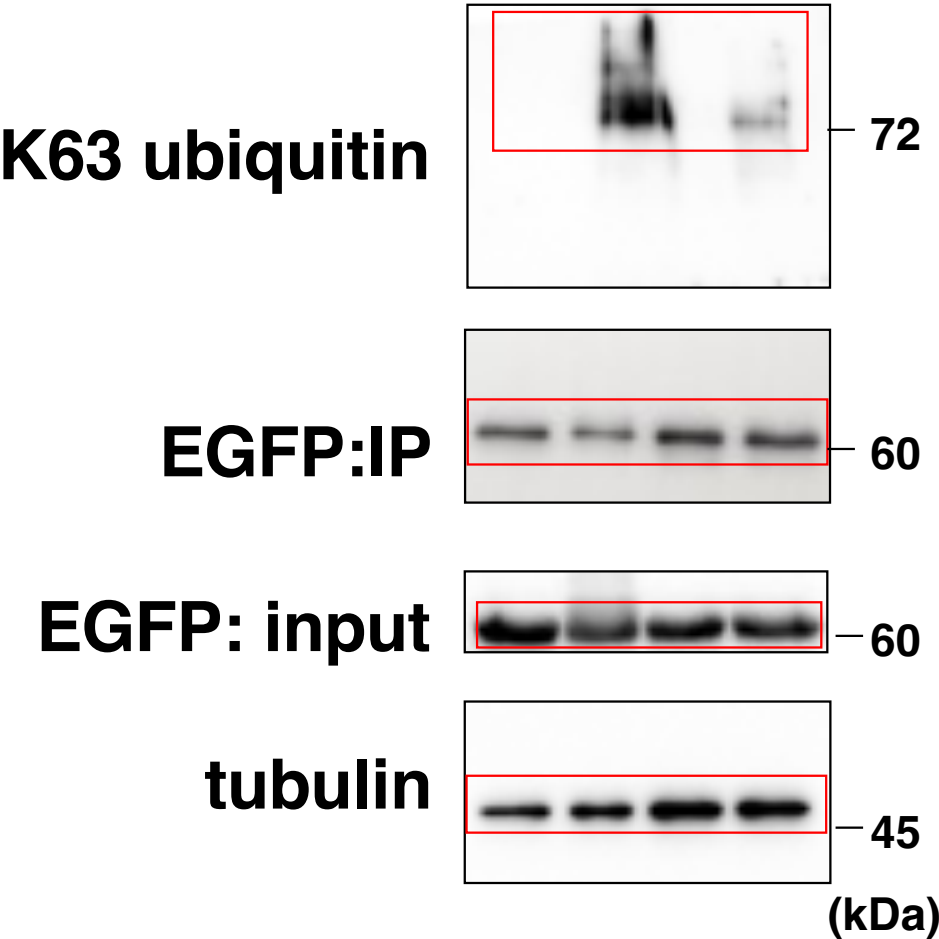

Supplement: Source Data Fig. 7 — Unprocessed western blots and/or gels. [file 41556_2023_1098_MOESM14_ESM.pdf]

**Fig. 8a**

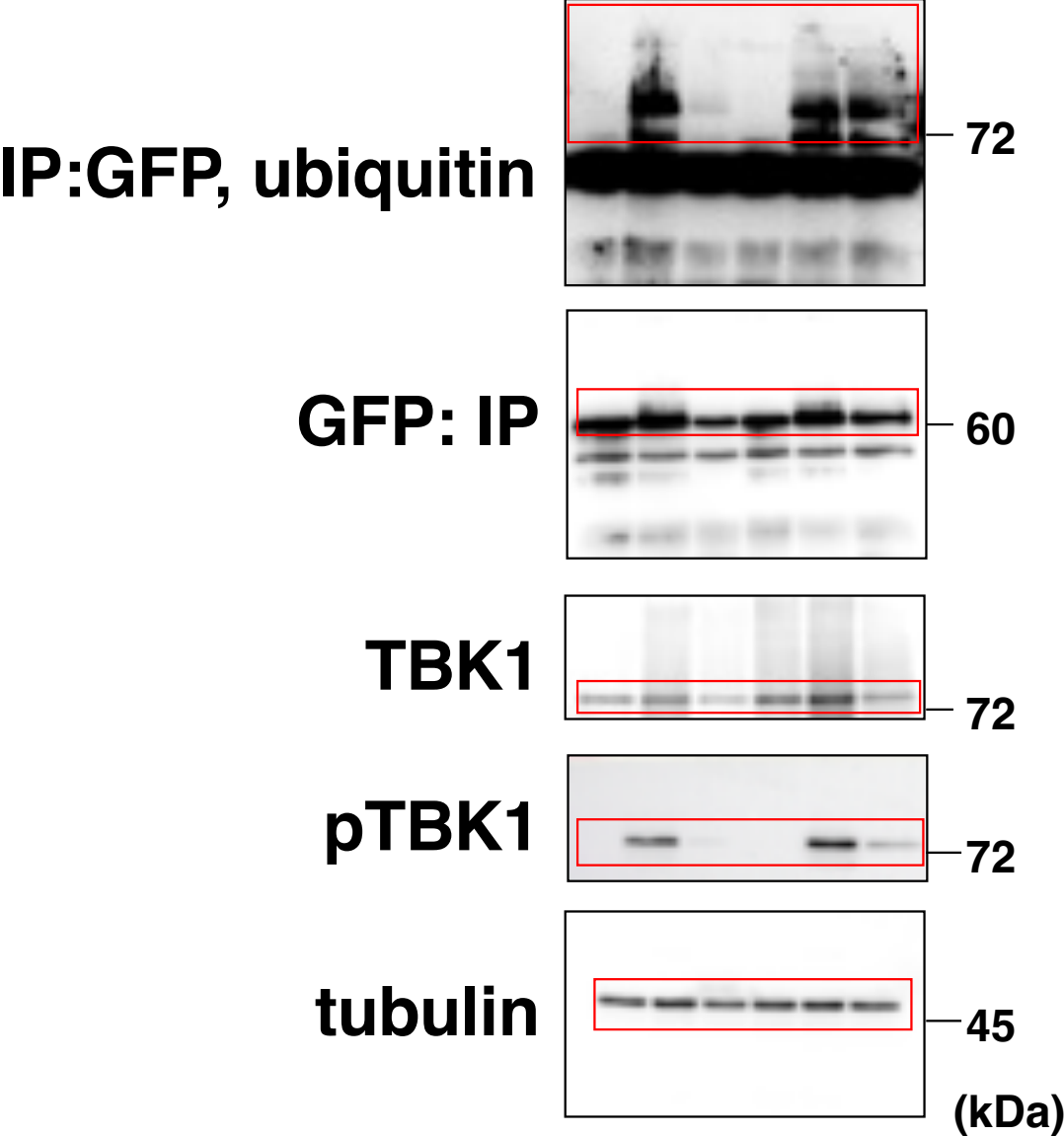

Supplement: Source Data Fig. 8 — Unprocessed western blots and/or gels. [file 41556_2023_1098_MOESM16_ESM.pdf]

**Ex Fig. 1f**

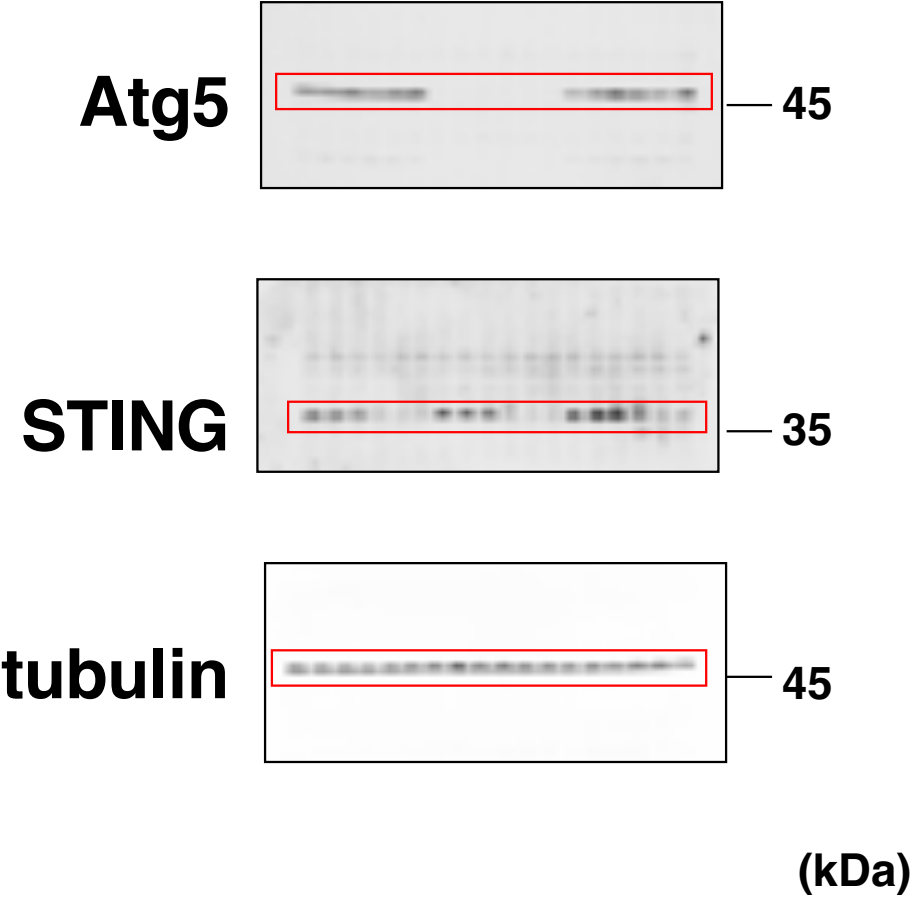

Supplement: Source Data Extended Data Fig. 1 — Unprocessed western blots and/or gels. [file 41556_2023_1098_MOESM18_ESM.pdf]

**Ex Fig. 2c**

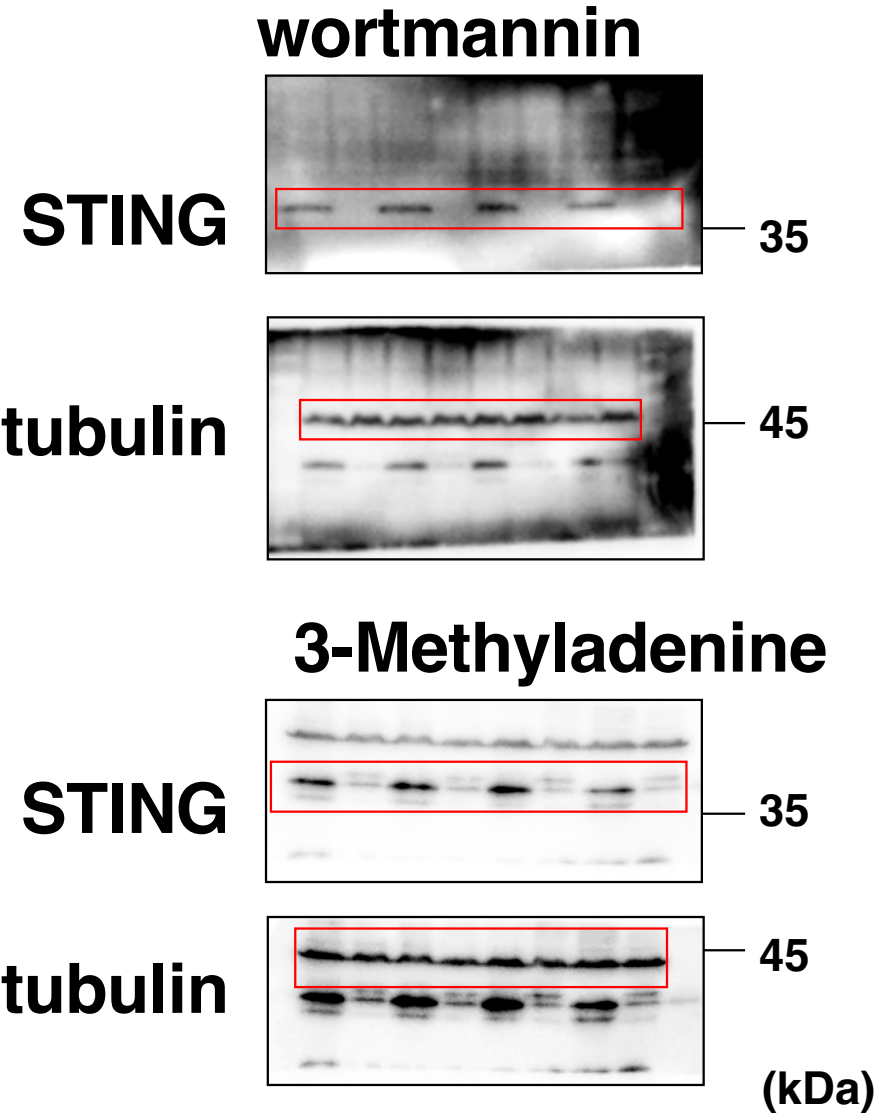

**Ex Fig. 2d**

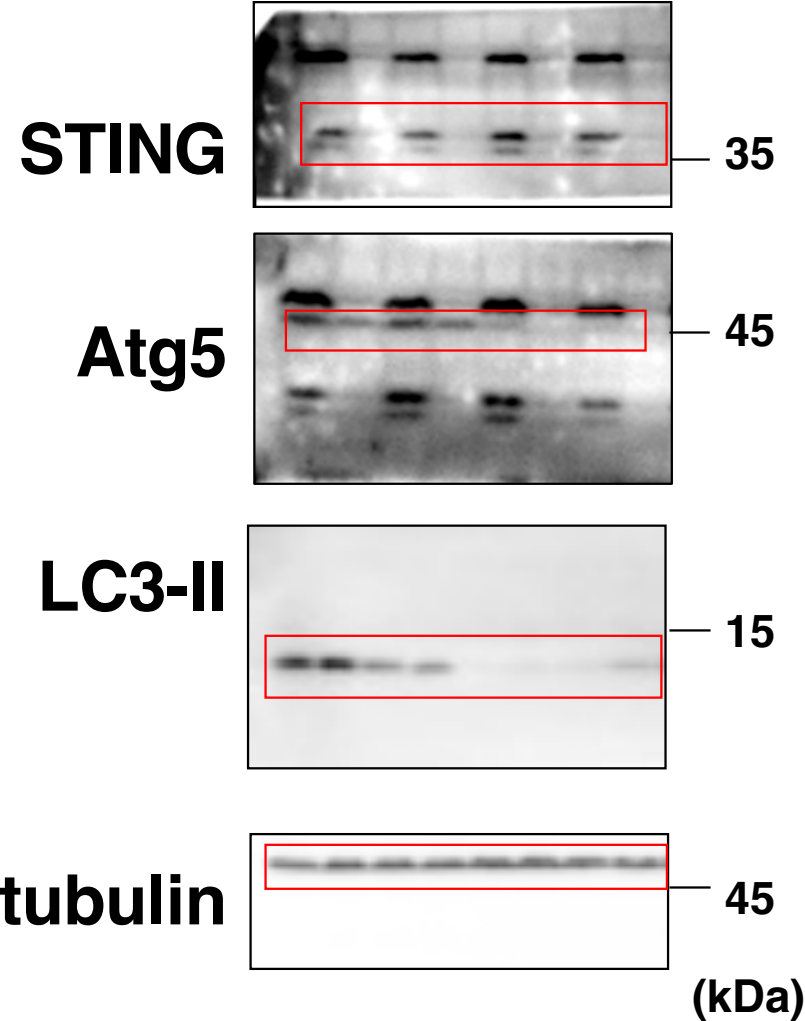

Supplement: Source Data Extended Data Fig. 2 — Unprocessed western blots and/or gels. [file 41556_2023_1098_MOESM19_ESM.pdf]

**Ex Fig. 10e**

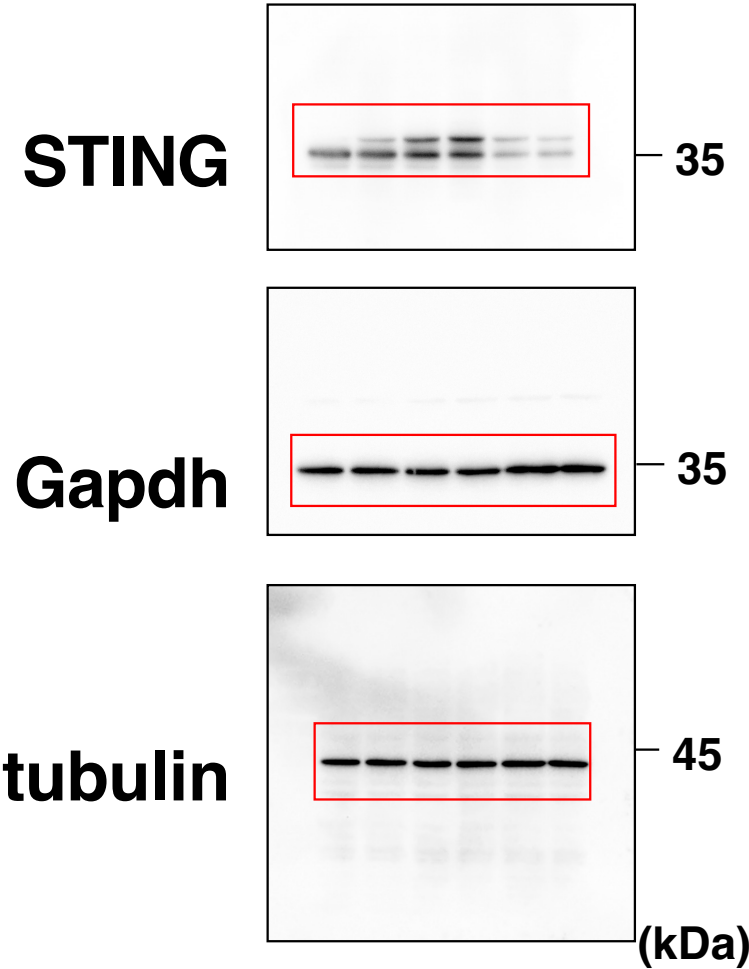

Supplement: Source Data Extended Data Fig. 10 — Unprocessed western blots and/or gels. [file 41556_2023_1098_MOESM26_ESM.pdf]
